# Author Correction: A Satellite Imagery Dataset for Long-Term Sustainable Development in United States Cities

**DOI:** 10.1038/s41597-023-02862-0

**Published:** 2023-12-21

**Authors:** Yanxin Xi, Yu Liu, Tong Li, Jingtao Ding, Yunke Zhang, Sasu Tarkoma, Yong Li, Pan Hui

**Affiliations:** 1 Department of Computer Science, University of Helsinki, Helsinki, Finland; 2 Beijing National Research Center for Information Science and Technology (BNRist), Beijing, P. R. China; 3 Department of Electronic Engineering, Tsinghua University, Beijing, P. R. China; 4 Computational Media and Arts Thrust, Hong Kong University of Science and Technology (Guangzhou), Guangzhou, P. R. China; 5 Division of Emerging Interdisciplinary Areas, Hong Kong University of Science and Technology, Hong Kong, P. R. China

**Keywords:** Sustainability, Socioeconomic scenarios, Sustainability, Socioeconomic scenarios

Correction to: *Scientific Data* 10.1038/s41597-023-02576-3, published online 04 December 2023

In this article the author name Jingtao Ding was incorrectly written as Jintao Ding. The original article has been corrected.

